# An AAM-Based Identification Method for Ear Acupoint Area

**DOI:** 10.3390/biomimetics8030307

**Published:** 2023-07-12

**Authors:** Qingfeng Li, Yuhan Chen, Yijie Pang, Lei Kou, Dongxin Lu, Wende Ke

**Affiliations:** 1 Health Management System Engineering Center, School of Public Health, Hangzhou Normal University, Hangzhou 311121, China; l20197092@hznu.edu.cn; 2 Department of Mechanical and Energy Engineering, Southern University of Science and Technology, Shenzhen 518055, China; 12132246@mail.sustech.edu.cn (Y.C.); 12232142@mail.sustech.edu.cn (Y.P.); 3 Institute of Oceanographic Instrumentation, Qilu University of Technology (Shandong Academy of Sciences), Qingdao 266075, China; koulei1991@qlu.edu.cn

**Keywords:** traditional Chinese medicine, ear image, identification, feature point, acupoint, segmentation, training set

## Abstract

Ear image segmentation and identification is for the “observation” of TCM (traditional Chinese medicine), because disease diagnoses and treatment are achieved through the massaging of or pressing on some corresponding ear acupoints. With the image processing of ear image positioning and regional segmentation, the diagnosis and treatment of intelligent traditional Chinese medicine ear acupoints is improved. In order to popularize ear acupoint therapy, image processing technology has been adopted to detect the ear acupoint areas and help to gradually replace well-trained, experienced doctors. Due to the small area of the ear and the numerous ear acupoints, it is difficult to locate these acupoints based on traditional image recognition methods. An AAM (active appearance model)-based method for ear acupoint segmentation was proposed. The segmentation was illustrated as 91 feature points of a human ear image. In this process, the recognition effects of the ear acupoints, including the helix, antihelix, cymba conchae, cavum conchae, fossae helicis, fossae triangularis auriculae, tragus, antitragus, and earlobe, were divided precisely. Besides these, specially appointed acupoints or acupoint areas could be prominent in ear images. This method made it possible to partition and recognize the ear’s acupoints through computer image processing, and maybe own the same abilities as experienced doctors for observation. The method was proved to be effective and accurate in experiments and can be used for the intelligent diagnosis of diseases.

## 1. Introduction

According to the traditional Chinese medicine system, the human ear is the place where the meridians of the human body converge, which can be helpful in disease diagnoses and treatment [1,2]. Modern medicine has also paid attention to the ear acupoints and systematic ear acupoint therapy for the efficient diagnoses and treatment of various diseases [3,4,5,6]. Obviously, ear image identification is the premise of disease diagnoses and treatment. Due to the large differences in the human ear in individuals and the dense acupoint area, there are difficulties in actual ear diagnosis. According to the most widely used Chinese national standard for ear acupoints, GB/T13734-2008, the segmentation of the auricular region area mainly focuses on dividing the anatomical structure of these acupoints, combining the naming and positioning of the acupoint area and the point. The partition of ear acupoints can be divided into nine large areas, namely the helix, antihelix, cymba conchae, cavum conchae, fossae helicis, fossae triangularis auriculae, tragus, antitragus, and earlobe. Herein, according to the respective standards, the helix can be divided into 12 areas, the antihelix into 13 areas, the concha auriculae (including the cymba conchae and cavum conchae) into 18 areas, the fossae helicis into 6 areas, the fossae triangularis auriculae into 5 areas, the tragus into 4 areas, the antitragus into 4 areas, and the earlobe into 9 areas [7], which can be distinguished by an orderly connection of 91 human ear acupoints scattered on the ears. According to the preliminary investigation, the current acupoint positioning is still in a very primitive operation stage, with many urgent problems needing to be solved:

(1) The location of the acupoints described in the traditional textbooks is not intuitive and clear;
(2) Personnel want to accurately locate, need a lot of training, and spend a lot of energy;
(3) It is difficult for non-professionals to find the right acupoints for daily healthcare;

Although acupoints have always been in the early practice mode, the medical value of TCM acupoints is recognized by the whole country and even the world, and has broad prospects, especially with regard to acupoint massages that can be carried out at home, the operation difficulty being low, and it being a very economical and convenient way of providing healthcare with good results. If the difficulty of finding acupoints can be reduced so that personnel without professional training can also find these acupoints very quickly and accurately and implement massages, it would be very beneficial for the popularization of traditional Chinese medicine acupoints and the development of traditional Chinese traditional medicine. The introduction of a computer to identify and display the ear image acupoint area and acupoints can not only effectively promote the development of traditional Chinese medicine, but is also a new application of computer vision in a new field.

At present, there have been relevant studies on the detection, normalization, feature extraction, and recognition of human external auricles. For example, Li Yibo et al. used the GVFsnake (gradient vector flow snake, GVFsnake) algorithm to automatically detect and segment external auricles [8]; Li Sujuan et al. proposed a normalization method for human ear images [9]; and Gao Shuxin et al. used the ASM (active shapemodel) algorithm to detect the contour of the outer ear [10].

Based on the ASM algorithm, Timothy F. Cootes et al. added a set of appearance models to form a relatively mature set of AAM algorithms for image identification [11]. The original AAM algorithm had a poor robustness and could not adapt to the interference of external environments. To solve the problem, E. Antonakos et al. combined the HOG (Histogram of Oriented Gradient) features with the AAM algorithm to reduce the impacts of light and occlusion on the recognition effect of the target images [12] and Lucas–Kanade was introduced into the search process of the AAM algorithm to improve the algorithm’s operation efficiency [13]. For images with complex feature point connections, the feature point identification of the AAM algorithm often requires many iterations and it is easy to fall into a local optimal solution; thus, the Gaussian–Newton optimization method [14] and Bayesian formula [15], etc., have been cited in the optimization process of the AAM algorithm, which improved its operation efficiency. In terms of application, researchers have mainly applied the AAM algorithm to face identification, which has obtained good results [16,17,18,19]. Chang Menglong et al. used the ASM algorithm to locate the acupoints that overlap with facial feature points [20]. Compared to the ASM algorithm, the AAM algorithm can identify and divide the ear point area more accurately and establish the contour of the object through the training set. Wang Yihui et al., based on the AAM algorithm, achieved the localization of the ear region in human ear images by connecting the feature points that make up the ear region separately [21].

The basic idea of the AAM algorithm is to divide the face image into two parts: shape and texture. By modeling the shape and texture, the recognition and tracking of the face can be achieved. A shape model is composed of a set of key points, while a texture model is composed of a set of feature vectors. Due to the characteristics of the AAM model, facial feature positions can be successfully detected. That is, the process of the AAM model matching can detect faces and facial features. Therefore, the AAM algorithm has been widely applied in both face detection and facial feature detection.

In order to improve the effective identification, segmentation, and feature point matching of the ear acupoints’ region images, and visually show the distribution of the hole area on the whole human ear, the AAM algorithm was used to obtain 91 feature points of a human ear image, in which the ear acupoints were identified, as well as the regions through the existing feature points. An ear region division method to visually represent the structure of an arbitrary ear was constructed. The ear area segmentation and appointed acupoint’s highlighting were achieved. The complete AAM-based method for ear image processing was applied in practical ear diagnoses.

## 2. AAM Algorithm Process

**Figure 1:**
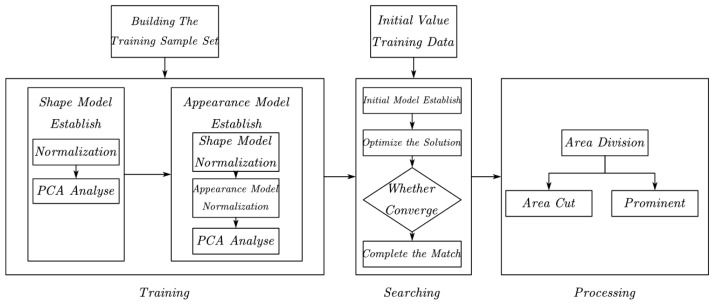
AAM algorithm process.

The implementation of the AAM algorithm on ear points is a tedious and comprehensive process, which not only includes the subject of the AAM algorithm, but also needs to introduce optimization-based feature point identification, experience-based acupoint adjustment, and position relationship calculation. The AAM includes image learning training and target image search identification, in which the learning model training process is mainly achieved through the input image and feature points to establish the image feature model, including the shape model and appearance model; the trained image and search process changes the average feature model through changing the direction of the existing model and obtaining a feature model that is mostly consistent with the target image. The flow chart is shown in Figure 1. The training process, the searching process of the AAM algorithm, and the display and segmentation of the ear acupoints’ area, etc., are presented in the following sections.

## 3. AAM Algorithm Training Process

### 3.1. The Establishment of Shape Model

The input feature points are processed and transformed to the form of the vector ***a_i_***, which is used to characterize the shape features of a picture:

(1) ai=(x1i,y1i,x2i,y2i,⋯,xN2i,yN2i),i=1,2,⋯,n

among which, *i* represents the serial number of an image, ***a_i_*** represents the characteristics of the picture of number *i*, *N*/*2* represents the number of the feature points, *x* and *y* represent the coordinates in the horizontal and longitudinal directions from the top left corner of the image, and *n* represents the total amount of training pictures.

(1) Normalization of shape model

The feature points are aligned to the average human ear model using the Procrustes method, where the corresponding shape vector ***a_i_*** for each image has four transformed degrees of freedom, two degrees of translation, a rotational degree, and a size-scaled degree. When these four degrees of freedom are successively expressed in the column vectors, the change relationship of the human ear shape vector of each training picture can be represented by a corresponding four-dimensional vector ***Z_i_***.

For ***E_i_*** = ***Z_i_^T^WZ_i_***, the normalization process can be converted into a minimization process of ***E_i_***, ***W*** is a diagonal matrix of order *N* in which *N* = 182, and the element *ω_i_* in the matrix satisfies:

(2) $$\omega_{k}={\left(\sum_{i=1}^{N}V_{R_{ki}}\right)}^{-1}$$

among which, *V_R_* represents the variance in the distance between point *k* and point 1 between different training samples.

(2) Principal component analysis (PCA)

Using a set of vectors ***a_i_*** normalized into a matrix ***A***, ***A*** is multiplied by its transposition to obtain the covariance matrix of the feature point position; the change law of the feature point position is as follows:

(3) $$S=\frac{1}{n}\sum_{i=1}^{n}{\left(a_{i}-\bar{a}\right)}^{T}\cdot (a_{i}-\bar{a})$$

The eigenvalues and eigenvectors of the acquisition ***S*** are (*λ*_1_, *λ*_2_,*…*, *λ_N_*) and (***n*_1_**, ***n*_2_**, …, ***n_N_***), respectively, and then the required shape model is:

(4) $$a=a_{avg}+\sum_{i=1}^{N}\lambda_{i}{\vec{n}}_{i}$$

among which, *a_avg_* is the initialized model after each alignment, ***n_i_*** (eigenvector) indicates each change and its direction of change, and *λ_i_* (eigenvalue) represents the weight of each change in the model.

### 3.2. The Establishment of Appearance Model

In addition to the shape model, the appearance model continues to be introduced and reflects the color change law around the formed area between any set of characteristic points, so that it can better adapt to the more complex image changes and shooting lighting conditions.

(1) Normalization of shape model

**Figure 2:**
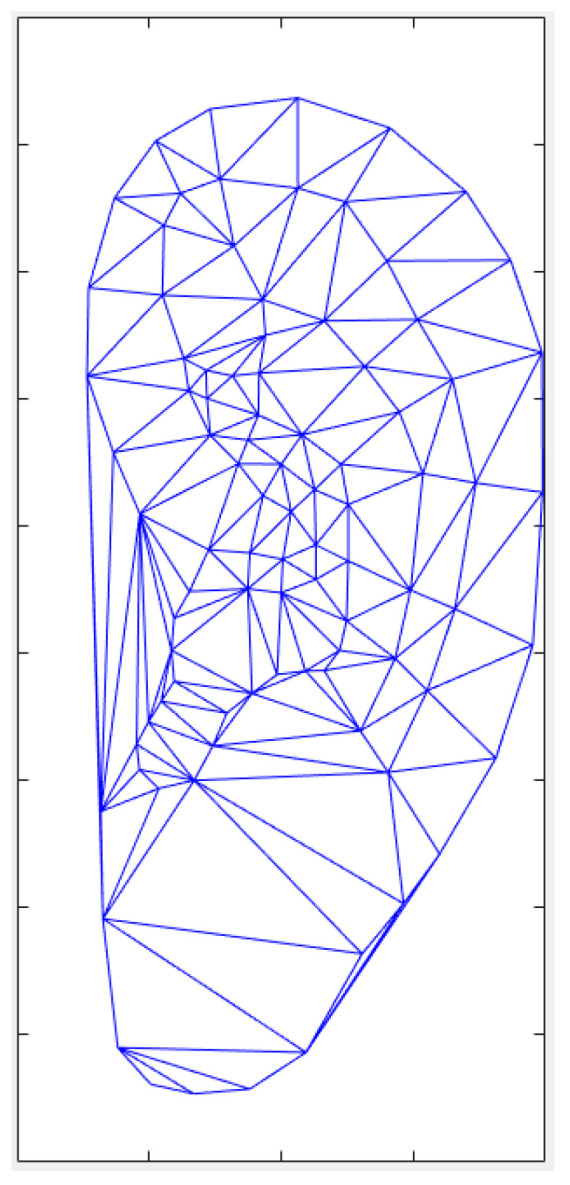
Ear model partition based on the Delaunay triangulation algorithm.

In order to facilitate the acquisition of the appearance model, a large shape needs to be divided into several small shapes to facilitate the calculation and storage. The Delaunay triangle algorithm is used to divide the large area of the human ear into several triangle regions [17]. The division effect is shown in Figure 2, and then the required set of triangles is obtained by artificially removing a small part of the redundant triangles.

The feature points are divided into several triangles and the shape image of the human ear is combined from these triangles. Therefore, adjusting the shape of a triangle can change the shape of the entire human ear, and transforming each triangle to the average shape of the entire human ear can obtain the average shape. When changing the substance of the triangle and the partial geometry relationship, as shown in Figure 2, any point ***p*** within the triangle will map to a new coordinate point ***p′*** in the new triangle, with their coordinates satisfying the relationship among the three triangular vertices, ***p*_i_**, ***p*_j_**, and ***p*_k_** (***p_i_′***, ***p_j_′***, and ***p_k_′***):

(5) $$p=\alpha p_{i}+\beta p_{j}+\gamma p_{k}$$

Since the coordinates of point ***p*** are known, the three vertex coordinates of the triangle before the deformation are known and the required new coordinate points can be obtained by obtaining their geometric relationship ***p′***. Letting any point of a coordinate be ***p*** = [*x*,*y*]*^T^*, the three vertex coordinates of the triangle are, respectively, ***p_i_*** = [*x*_i_,*y*_i_]*^T^*, ***p*_2_** = [*x*_j_,*y*_j_]*^T^*, and ***p*_3_** = [*x*_k_,*y*_k_]*^T^*, the positional relationship of ***p*** as well as ***p*_1_**, ***p*_2_**, and ***p*_3_** are calculated, and the values of *α*, *β*, and *γ* are, respectively,

(6) $$\alpha =\frac{-(x-x_{j})(y_{k}-y_{j})+(y-y_{j})(x_{k}-x_{j})}{-(x_{i}-x_{j})(y_{k}-y_{j})+(y_{i}-y_{j})(x_{k}-x_{j})}$$

(7) $$\beta =\frac{-(x-x_{k})(y_{i}-y_{k})+(y-y_{k})(x_{i}-x_{k})}{-(x_{j}-x_{k})(y_{i}-y_{k})+(y_{j}-y_{k})(x_{i}-x_{k})}$$

(8) γ=1−α−β

The above process shows how to realize the partial linear affine. The established sample model can be sampled in the triangle area of the average shape model, so that the construction of the appearance model cannot be affected by the shape model and realize the normalization of the shape model.

(2) Normalization of appearance model

In Equation (1), after normalization, the shape model and the transformation of the human ear image under the average shape model are obtained. To convert the image information into vectors, the gray values of each pixel point are arranged in a set of vectors in a fixed order. ***g*** = (*g*_1_, *g*_2_, *g*_3_, …*g_n_*), where *n* is the number of all the pixels in a shape-independent texture image. In order to overcome the impact of different overall illuminations, the shape-independent grayscale vector needs to be normalized. The so-called normalization of the gray vector is to generate a gray vector with a mean of 0 and variance of 1.

Upon normalizing the vector ***g***, a more canonical texture sample vector can be obtained ***g′***, and the specific implementation of the normalization is [16]:

(9) $$g^{\prime }=\left(\frac{g_{1}-m}{\sigma },\frac{g_{2}-m}{\sigma },\frac{g_{3}-m}{\sigma },\cdots ,\frac{g_{4}-m}{\sigma }\right)$$

In order to achieve the normalization of all the elements in *g′* that have a mean of 0 and varying variances of l, *σ* is the scaling factor that is the variance of *g_i_* and *m* is the displacement factor that is the mean of *g_i_*, thus:

(10) $$\begin{array}{l} m=\frac{1}{n}\sum_{i=1}^{n}g_{i}\\ \sigma =\sqrt{\frac{1}{n}\sum_{i=1}^{n}{\left(g_{i}-l\right)}^{2}} \end{array}$$

(3) Principal component analysis (PCA)

After listing the pixel information of each point in the region as a vector, the average appearance model is desired, which is the aligned initial model and corresponding transformation direction. The method is as same as the PCA analysis of the shape model.

(11) $$A=A_{avg}+\sum_{i=1}^{n}{\lambda^{\prime }}_{i}{{\vec{n}}^{\prime }}_{i}$$

among which, *A_avg_* is the initialization of the model for each alignment, ***n****′_I_* (eigenvector) indicates each change and its direction, and *λ′_I_* (eigenvalue) represents the weight of each change in the model.

## 4. AAM-Based Searching Process

### 4.1. Determination of the Initial Value of Feature Points

**Figure 3:**
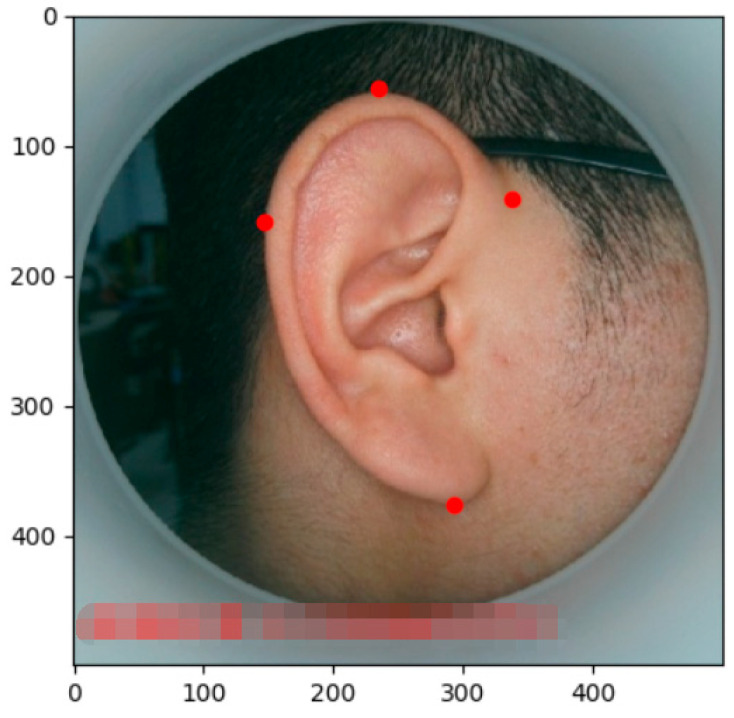
Example of the human ear boundary calibration.

In the calibration of the boundaries for the existing images, shown in Figure 3, the process obtains the coordinates of the four points shown as red points for each image as the corresponding position information of the human ear during the image.

The interception of a part of a human ear image zone from the existing image as a negative sample eliminates the effect on the initial position of the part with similar grayscale values of the image to be tested. The above pictures and data are trained using the HOG (Histogram of Oriented Gradient) characteristics and SVM (Support Vector Machine), and then processed through the image to be tested after the training. The data can roughly determine the position and size of the human ear in the image to be tested. Then, the mean shape model obtained during the AAM training process is replaced with the specified position and obtains the initial value of the search process.

### 4.2. AAM Algorithm Search Process

During the search process of the AAM algorithm, we use the optimal solution scheme of Lucas–Kanade + Inverse Compositional (IC) [11]. After the two models are established, the weights of each change are changed to generate various pictures. The feature points of the target are obtained by seeking the optimal solution during *λ_i_* in the shape model and *λ′_I_* in the appearance model. The functions used for the optimization solution are as follows [11]:

(12) $$\left\{\begin{array}{c} \underset{\Delta \lambda }{\arg\min}{\left\|t(\omega (\lambda ))-a_{\lambda^{\prime }}(\omega (\Delta \lambda ))\right\|}_{I-U_{A}U_{{}_{A}}^{T}}^{2}\\ \underset{\Delta \lambda^{\prime }}{\arg\min}{\left\|t(\omega (\lambda ))-a_{\lambda^{\prime }+\Delta \lambda^{\prime }}(\omega (\Delta \lambda ))\right\|}^{2} \end{array}\right.$$

The first function is the optimization solution of the shape weight change (Δ*λ*) when the appearance weight is invariant, *t* indicates the test image, and *ω*(*λ*) refers to the pair transforming the image within the shape region at *p* onto the average shape model; thus, *t*(*ω*(λ)) is the distortion of the test image. *A_λ’_* represents the texture model under the *λ′* condition, *ω*(Δ*λ*) refers to the distortion model after a small deformation, and the optimal solution of the two difference square error represents the optimal change step length (Δ*b*) of the shape change in a certain texture situation.

The problem can be simplified to a linear least-squares problem as:

(13) $$\Delta \lambda =H^{-1}J_{AIC}^{T}[t(\omega (\lambda ))-\bar{a}-U_{A}\lambda^{\prime }]$$

(14) $$\Delta \lambda^{\prime }=U_{A}^{T}[t(\omega (\lambda ))-\bar{a}(\omega (\Delta \lambda ))-U_{A}(\omega (\Delta \lambda ))\lambda^{\prime }]$$

among which, *J_AIC_* and *H*^−1^ are the Jacobian Matrix and Hessian Matrix obtained by a subset of eigenvalues *U*_A_, respectively.

**Figure 4:**
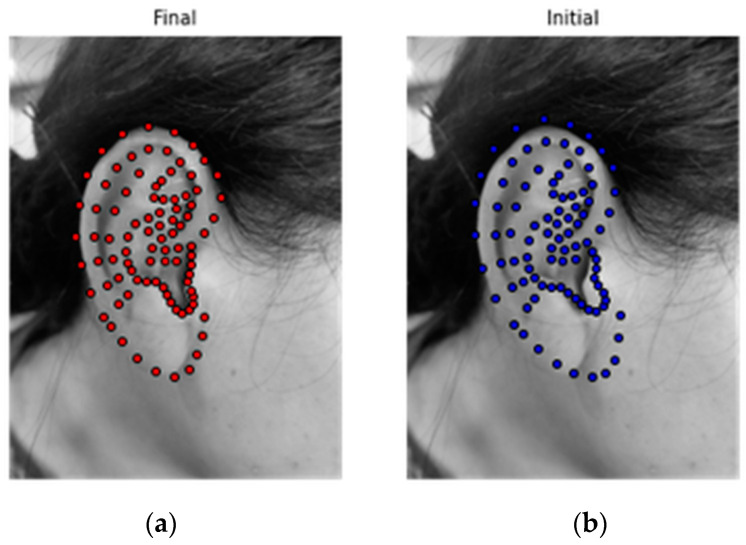
Results of the feature points; (**a**) final solution result; and (**b**) initial shape model.

The final solution of the feature points is obtained by multiple iterations of the above equations, so an accurate positioning of the 91 feature points of the target image is enabled. The initial shape output effect and final solution results are shown in Figure 4, where the dots represent the characteristic points.

## 5. Reformation and Division of Feature Points of Ear Acupoints

### 5.1. Earlobe Feature Point Reconstruction

The first nine feature points P^o^_1_–P^o^_9_ are connected by using straight lines, P^o^_2_P^o^_1_ are reversely extended at P^o^_0_, P^o^_8_P^o^_9_, as for the two adjacent points P^o^_n_ and P^o^_n+1_, the angular bisectors of ∠P^o^_n−1_P^o^_n_P^o^_n+1_ and ∠P^o^_n_P^o^_n+1_P^o^_n+2_ are crossed at *O_i_*, respectively, and the polar coordinate system is constructed taking *O_i_* as the origin. P^o^_k_ is the interpolation between P^o^_n_ and P^o^_n+1_ and the polar coordinates of the worthwhile point P^o^_k_ meet:

(15) $$\left\{\begin{array}{c} \theta_{k}=\frac{k}{k_{n}}(\theta_{P_{n+1}^{o}}-\theta_{P_{n}^{o}})+\theta_{P_{n}^{o}},\;k=1,2,\ldots k_{n-1}\\ R_{k}-R_{P_{n}^{o}}=\frac{\theta_{k}-\theta_{P_{n}^{o}}}{\theta_{P_{n+1}^{o}}-\theta_{P_{n}^{o}}}\cdot (R_{P_{n+1}^{o}}-R_{P_{n}^{o}}) \end{array}\right.$$

among which, *k* represents the serial number of the interpolation point, *k_n_* represents the number of interpolation points, and (*R_k_*, *θ_k_*) is the polar coordinate of the interpolation point.

**Figure 5:**
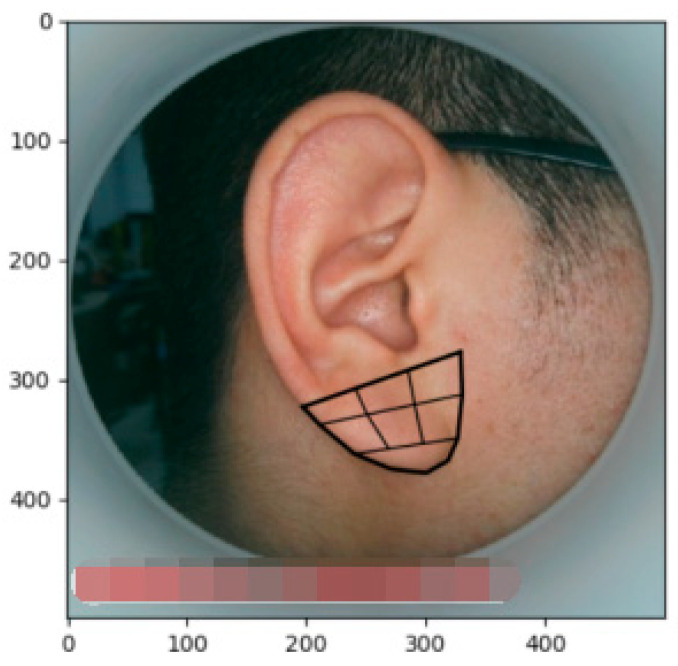
Earlobe characteristic points before the reconstruction.

**Figure 6:**
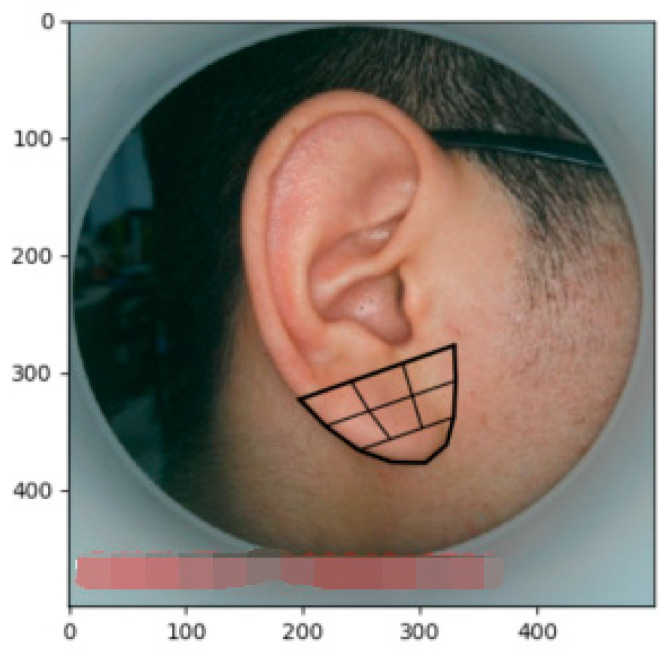
Reconstructed earlobe feature points.

All interpolation points are connected, letting P_1_ = P^o^_1_, P_9_ = P^o^_9_ link P_1_P_9_. The furthest point is taken from the P^o^_1_P^o^_9_ to P^o^_k1_ as *h*, letting P_5_ = P^o^_k1_. The parallel lines P_2_P_8_ and P_3_P_7_ cross the line at the points P_2_, P_8_, P_3_, and P_7_, respectively, so there are P_1_P_9_//P_2_P_8_//P_3_P_7_ and the distances to P_5_ are 2*h*/3 and *h*/3, respectively. The valent point of the length is taken from P_3_P_5_, P_5_P_7_:P_4_, P_6_. Finally, the nine characteristic points P_1_–P_9_ of the reconstructed earlobe can be obtained, and the position pairs before and after the point reconstruction are shown in Figure 5 and Figure 6.

### 5.2. Ear Acupoints’ Division

**Figure 7:**
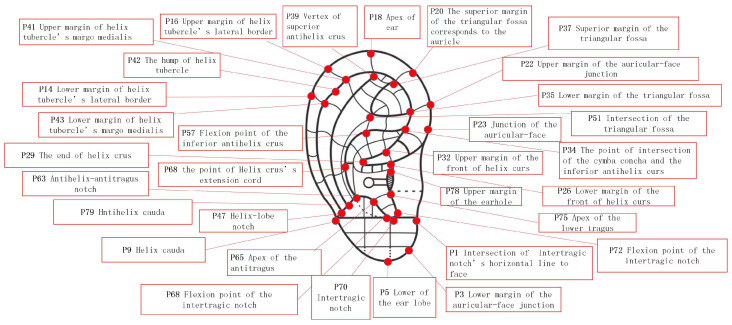
The division of human ear acupoint area under the national standard.

Through the existing characteristic points, according to the national standard GB/T13734-2008, the reference figure is shown in Figure 7.

However, in the national standard, the intersection point of the earlobe minimum point of the line section *P*_1_*P*_9_ needs to meet the third-class dividing point of the line section, which has certain requirements for the structure of the human ear itself. To satisfy the human ear specificity, the tertiary points of the line segments *P*_1_*P*_9_, *P*_2_*P*_8_, and *P*_3_*P*_7_ are usually taken for connection.

### 5.3. Main Area Segmentation

**Figure 8:**
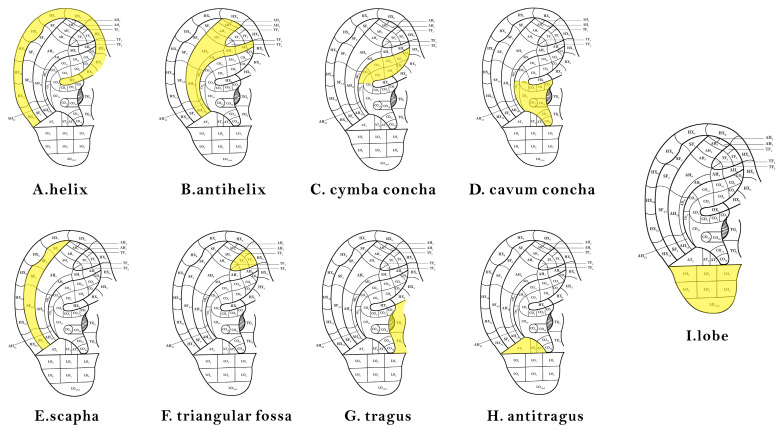
Locations of each major area.

To meet the diagnostic needs of the ear acupoints, there are nine more representative areas of the human ear (shown in Figure 8): the helix, antihelix, cymba conchae, cavum conchae, fossae helicis, fossae triangularis auriculae, tragus, antitragus, and earlobe. Divided separately, these also lay the foundation for the later identification of the corresponding diseases through the ear acupoints’ region vision.

**Figure 9:**
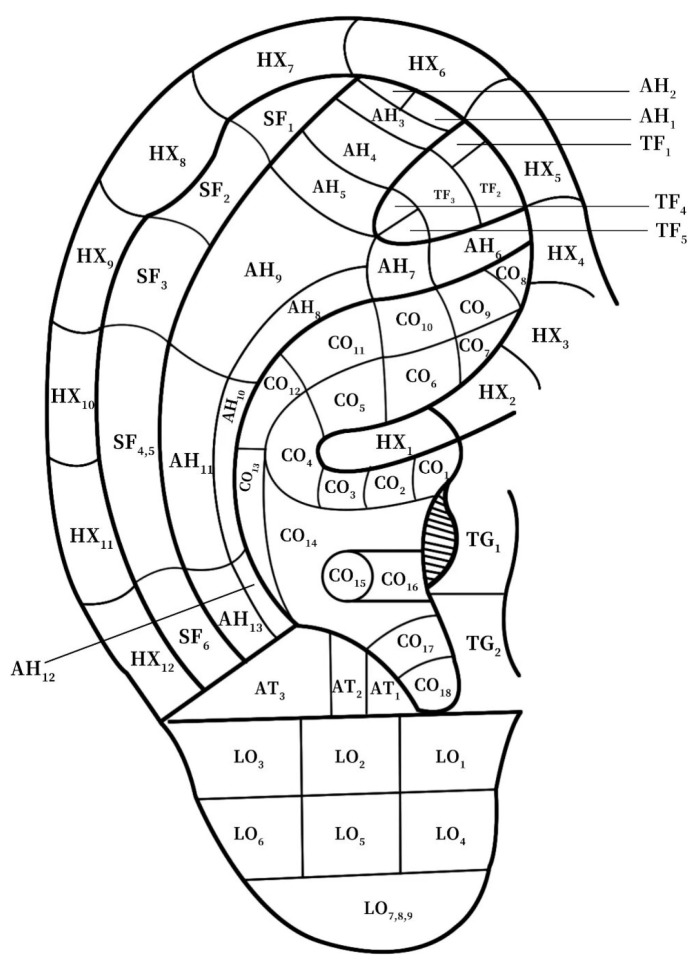
Distribution of acupoints in earrings (front).

In actual diagnosis and treatment, it is usually necessary to obtain a specific ear acupoint efficiently. Therefore, for a specific acupoint, the corresponding positions in the ear image need to be highlighted. The distribution of the human ear acupoints are shown in Figure 9, in the Chinese national standard GB/T13747-2008.

## 6. Experiments

### 6.1. Data Sources

**Figure 10:**
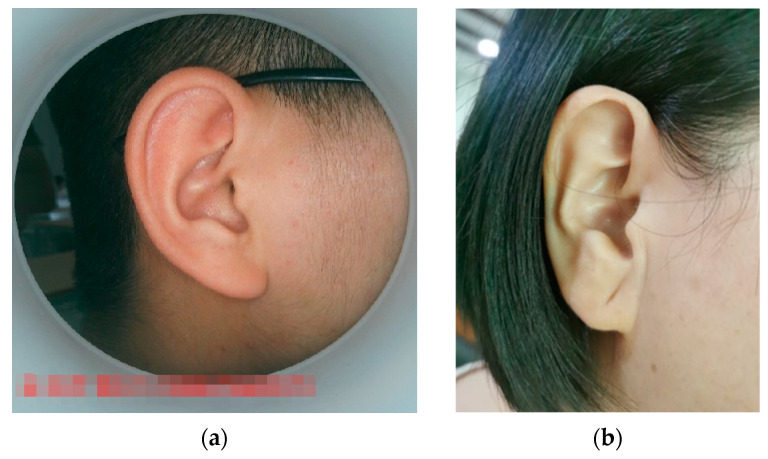
Example of training sample: (**a**) standardized training samples. (**b**) Mobile phone training samples.

The sample images trained in this experiment have two sources. Firstly, 68 standardized images were collected by the hospital using professional equipment, the image sizes were unified to 500 × 500 pixels, and, at the same time, these ear images were obtained using the same light conditions and same angles. Secondly, 100 unstandardized images were taken by mobile phones at the Mishixiang Community Health Service Center, Hangzhou City, Zhejiang Province, and these images were in different shapes and different shooting conditions. Some of the sample images are shown in Figure 10.

Referring to the latest national standard of auricular acupoints, GB/T13734-200891, in order to achieve a more accurate positioning and segmentation of each acupoint on the ear image, it was necessary to obtain 91 characteristic points on the human ear. The above 168 image samples were manually annotated to obtain the data of the respective 91 feature points to form the input sample set.

In addition, 500 pictures containing human ears were captured through cameras in medical centers and then used for upper, lower, left, and right boundary calibration, and some negative samples needed to be intercepted as the initial value training data of the hog feature combined with the SVM support vector machine.

### 6.2. Experimental Environment

The Menpo on Linux was constructed. The obtained training sample data and detected pictures were imported into the system to obtain the 91 feature points of the ear image for measurement. The 91 feature points were processed and conducted with a certain connection and filling operation to achieve the effect of the ear acupoints’ division and segmentation.

### 6.3. Experimental Result

**Figure 11:**
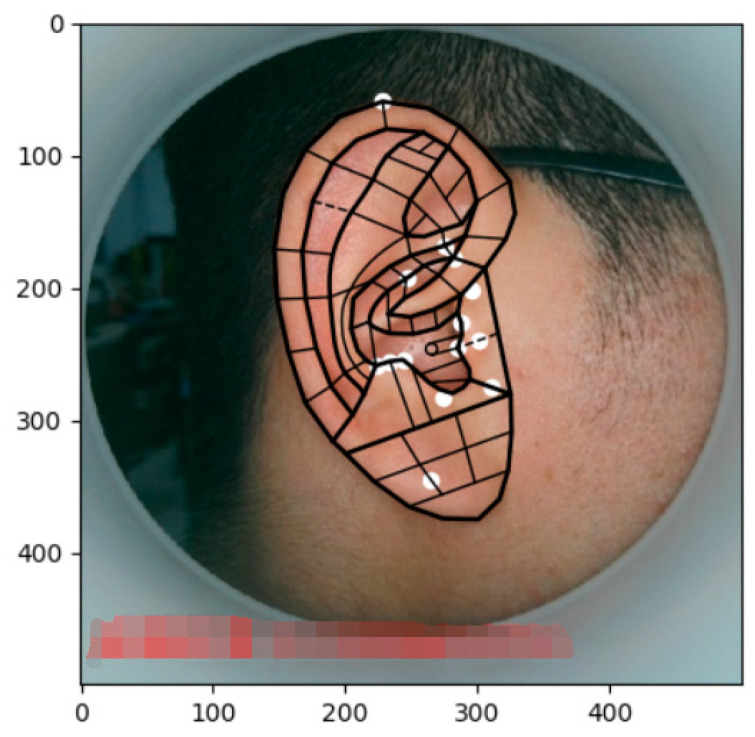
The division result of the ear acupoints.

The result of the final ear acupoints’ division is shown in Figure 11, where the white dots represent some representational acupoints. The method presented in this paper achieved the accurate positioning of the 91 feature points, the convergence accuracy rate was 100% under the standard equipment situation (the evaluation criteria for the success rate were from the acceptable number considered by experts divided by the total number of tests), and the average pixel error of the calibration point was less than six pixels. Therefore, the proposed method was far beyond existing earhole partition algorithms, both in terms of its localization accuracy and convergence accuracy. The success rate of the characteristic points’ identification for the ear photos taken by mobile phone, which were affected by an unstable light environment, could still be more than 95%.

**Figure 12:**
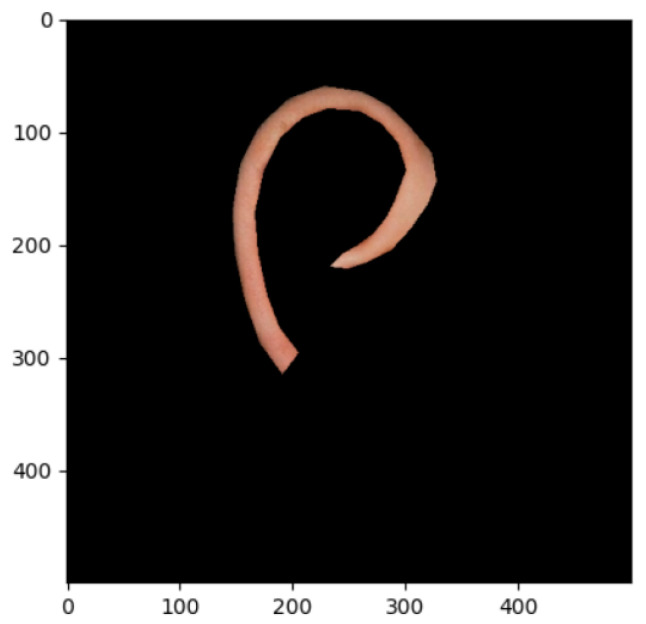
Helix segmentation.

**Figure 13:**
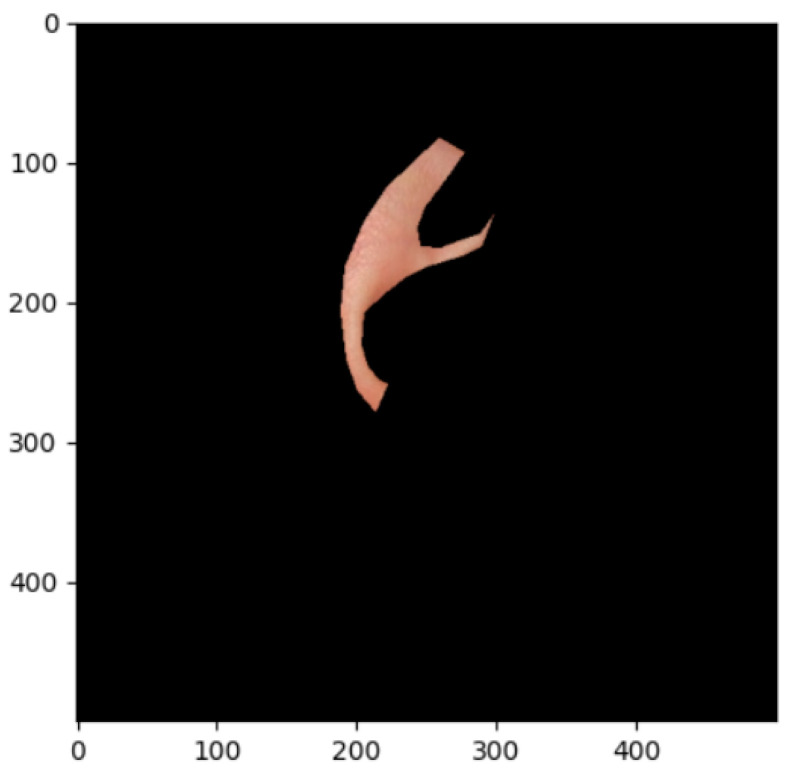
Antihelix segmentation.

**Figure 14:**
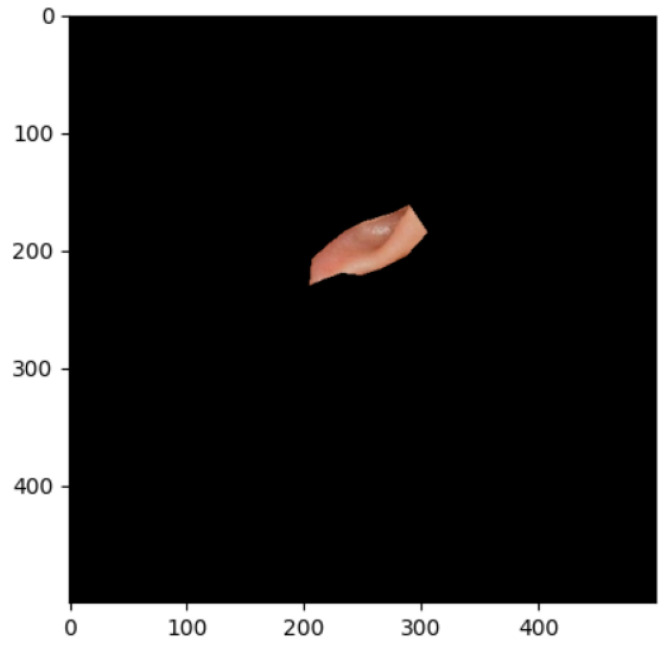
Cymba conchae segmentation.

**Figure 15:**
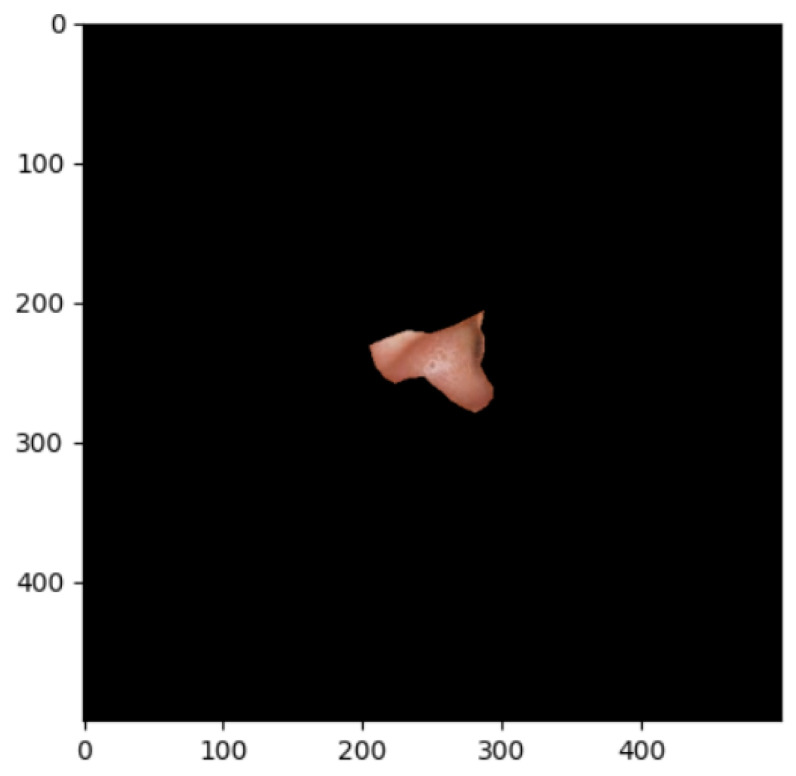
Cavum conchae segmentation.

**Figure 16:**
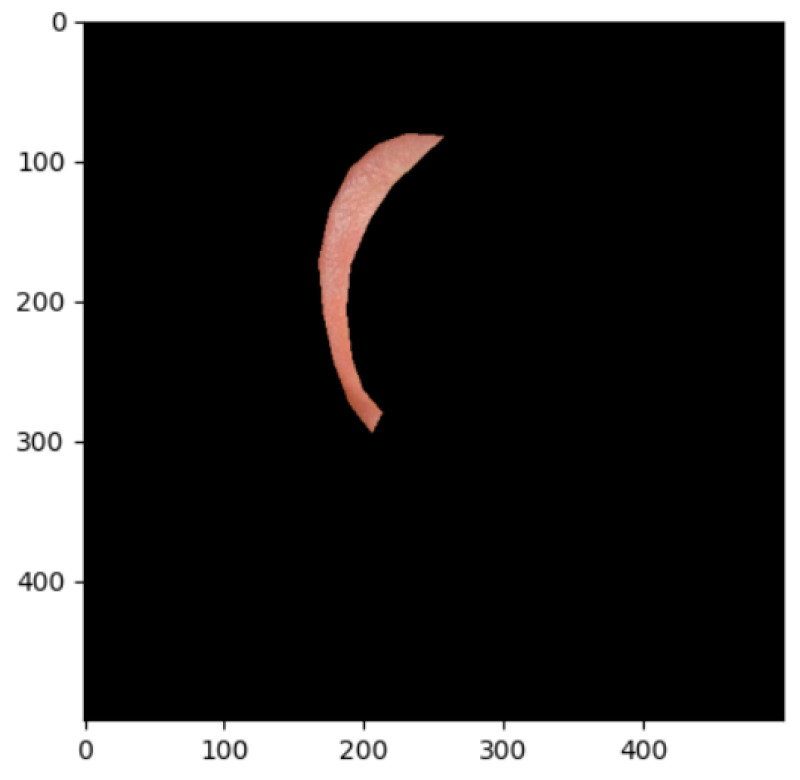
Fossae helicis segmentation.

**Figure 17:**
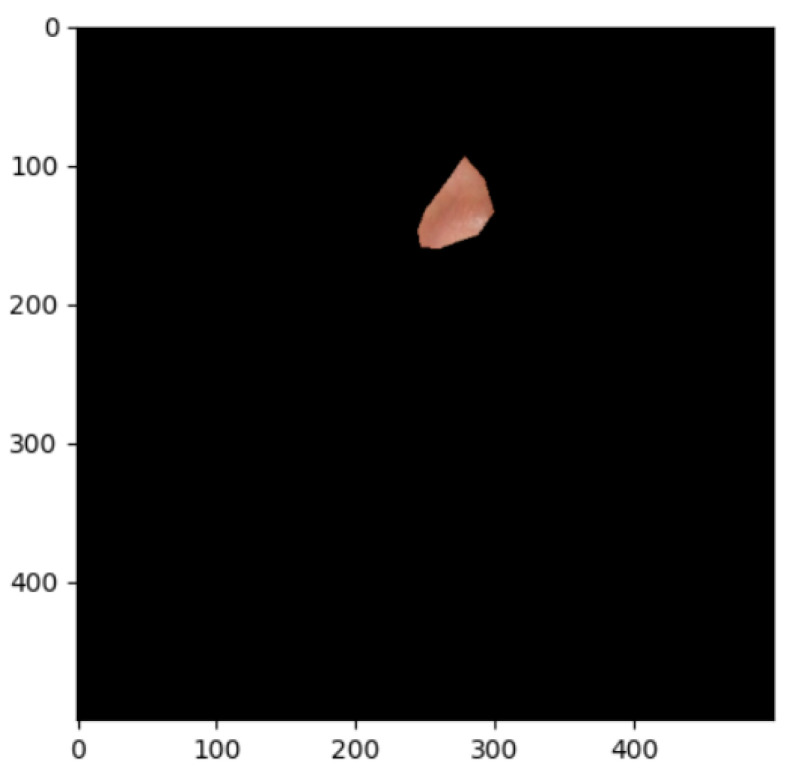
Fossae triangularis auriculae segmentation.

**Figure 18:**
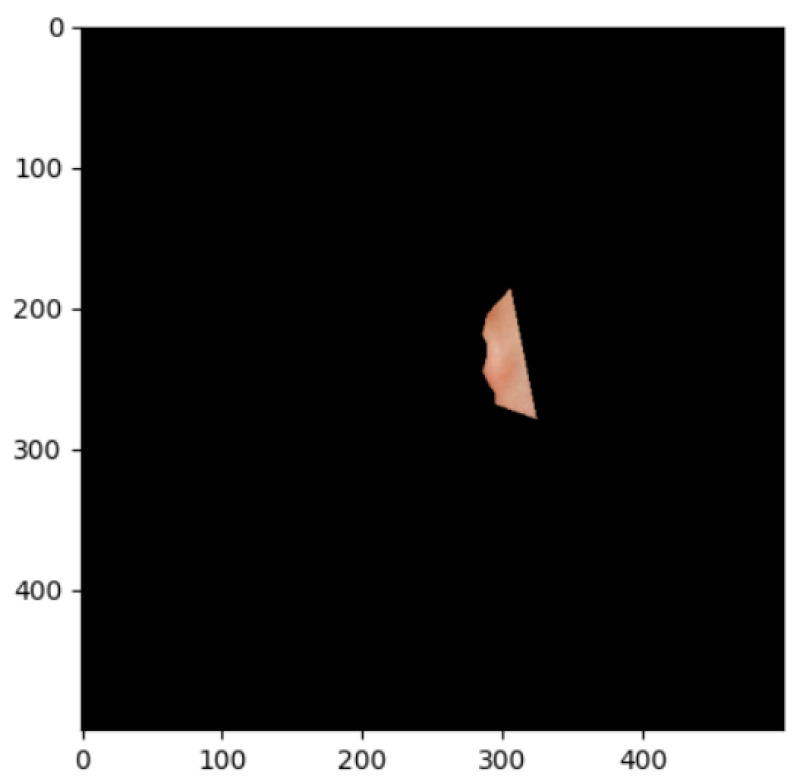
Tragus segmentation.

**Figure 19:**
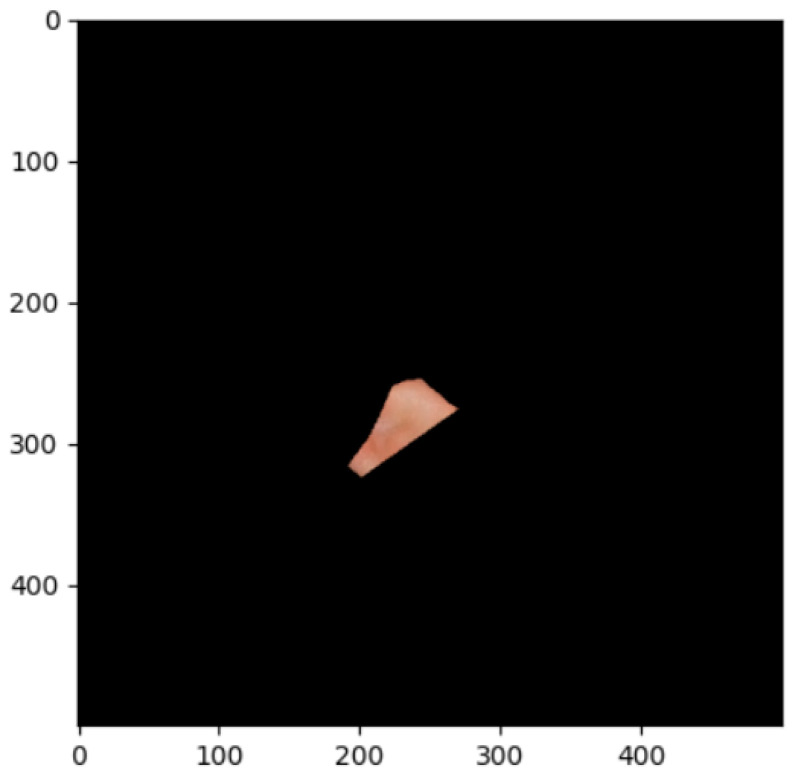
Antiragus segmentation.

**Figure 20:**
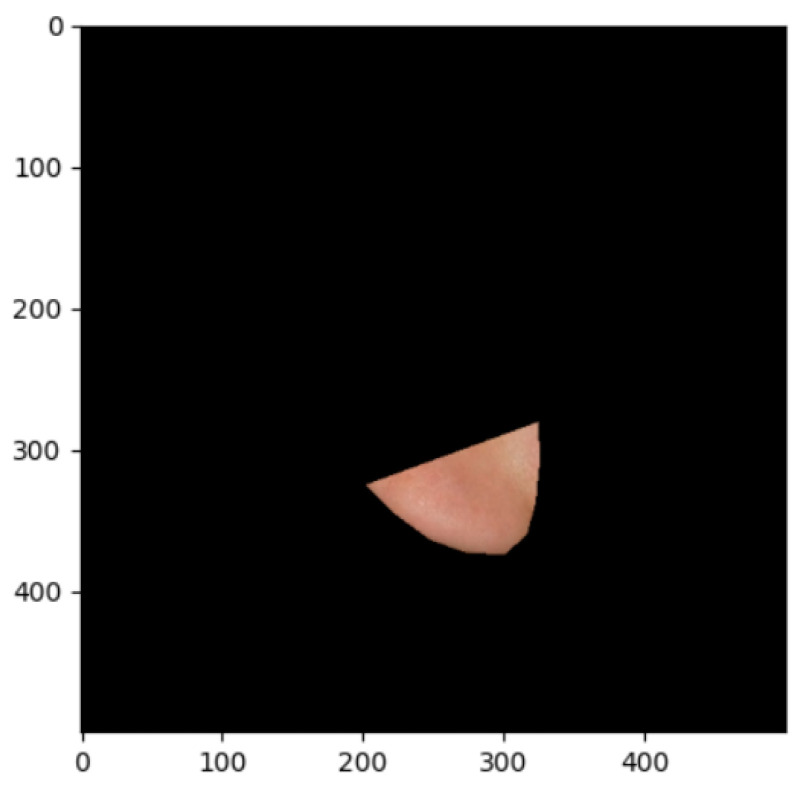
Earlobe segmentation.

The segmentation effects of the auricular regions are shown in the following Figure 12, Figure 13, Figure 14, Figure 15, Figure 16, Figure 17, Figure 18, Figure 19 and Figure 20. The helix segmentation is shown in Figure 12, antihelix segmentation is shown in Figure 13, cymba conchae segmentation is shown in Figure 14, cavum conchae segmentation is shown in Figure 15, fossae helicis segmentation is shown in Figure 16, fossae triangularis auriculae segmentation is shown in Figure 17, tragus segmentation is shown in Figure 18, and the antitragus and earlobe were divided precisely, as shown in Figure 19 and Figure 20, respectively. According to the figures, our proposed method was able to segment the target auricular regions accurately and there is no doubt that these segmentation works are going to make contributions to disease diagnoses. Compared to the experimental results in reference [20], our ear acupoint region recognition effect is clearer and more accurate, especially for the automatic segmentation of edge regions, which had a better performance.

**Figure 21:**
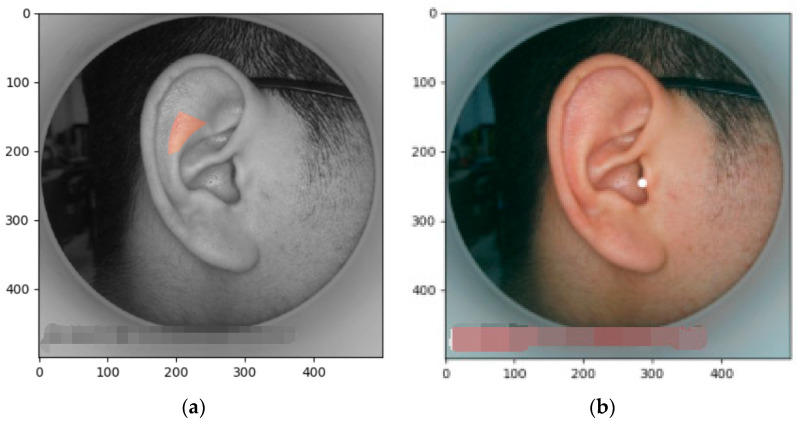
Renderings of prominent acupoints: (**a**) regional calibrated acupoint (input “Lumbosacral vertebrae”); and (**b**) point-calibrated acupoint (input “Adrenal gland”).

The partial results of the highlighted acupoints are shown in Figure 21, with the inputs “Lumbosacral vertebrae” and “Adrenal gland”.

The experiment results showed better processing. This was because the AAM algorithm promoted accuracy in its good recognition and tracking ability for different ear images, which can handle problems such as ear image deformation and posture. The AAM algorithm also has a fine resolution, which can express the features of the target in detail through the effects of shape and texture modeling.

## 7. Conclusions

This paper mainly studied the positioning and display methods of various acupoints and acupoint areas based on the AAM algorithm. Based on the AAM, the shape models and appearance models of the human ear were constructed, 91 feature points of the target ear image were extracted, and the acupoints’ area division was completed, as well as the segmentation extraction of 9 representative regions of the human ear and the display of each ear acupoint through the obtained 91 feature points. The research is very useful for graph-based ear acupoint positioning, promotes the development of ear acupoint diagnoses and treatment in smart Chinese medicine, and is helpful for the subsequent ear acupoint regional vision identification of corresponding diseases. In fact, the main drawbacks of the AAM algorithm are that it is sensitive to factors such as changes in lighting and facial expressions, requires post-processing, and does not have a very good average detection speed and real-time tracking. Furthermore, on some occasions, the annual calibration of the anatomical structure of the auricle may lead to a low accuracy in some ear point segmentations. In future work, we will collect images for constructing a larger dataset, including ear images and tongue images, etc., to expand the acupoint segmentation and recognition of a range of faces, introducing deep learning to make the speed of the acupoint area identification and segmentation faster. We hope that this AAM-based identification method will be applied to ear diagnoses and treatment, which play roles in practical medical engineering works.

## Data Availability

The data that support the findings of this study are available on request from the corresponding author.

## References

[1] Pu C., Bi H., Jin D., Shao M., Zhang Q., Zhang N. (2020). Meta-analysis of systematic evaluation of ear acupoint therapy for type 2 diabetes. Chin. J. Gerontol..

[2] Wang S., Han F. (2021). A Meta Analysis of Auricular Acupoint Therapy for Treating Tic Disorder. J. Tradit. Chin. Med. Univ. Hunan.

[3] Bu G., Lu M., Huang S. (2021). Effect of ear acupoint therapy on cognitive impairment in hypertensive patients with Tanshi Yongsheng type. China Mod. Dr..

[4] Chen W., Ding W., Li B., Zhang X. (2021). Reevaluation of systematic evaluation of ear acupoint therapy for primary hypertension. Chin. J. Integr. Med. Cardio/Cerebrovasc. Dis..

[5] Qian J., Wang Y., Wang Q., Li Y., Li M., Xu W. (2021). Acupoint selection rules of auricular therapy in treating constipation based on data mining. J. Clin. Med. Pract..

[6] Tian X., Sun Z. (2020). Application of auricular point therapy in assisted reproductive technology. Chin. J. Hum. Sex..

[7] Jiang Y. (2012). Auricular Therapy Combined with Danggui Yinzi in Treatment of Blood-Deficiency Wind-Attact Pattern Pruritus. Ph.D. Thesis.

[8] Li Y., Huang Z., Zhang H., Mu Z. (2010). An automatic ear detection method based on improved GVF Snake. Pattern Recognit. Artif. Intell..

[9] Li S.J., Feng J., Niu J.C. (2008). The research of the impact of normalization for feature extraction and recognition on ear biomitrice recognition. Comput. Knowl. Technol..

[10] Gao S.X., Mu Z.C. (2008). On image normalization in ear recognition. Control. Eng. China.

[11] Cootes T.F., Edwards G.J., Taylor C.J. (2001). Active Appearance Models. IEEE Trans. Pattern Anal. Mach. Intell..

[12] Antonakos E., Alabort-i-Medina J., Tzimiropoulos G., Zafeiriou S. HOG Active Appearance Models. Proceedings of the 2014 IEEE International Conference on Image Processing (ICIP).

[13] Antonakos E., Alabort-i-Medina J., Tzimiropoulos G., Zafeiriou S. (2015). Feature-based Lucas-Kanade and Active Appearance Models. IEEE Trans. Image Process..

[14] Tzimiropoulos G., Pantic M. Gauss-Newton Deformable Part Models for Face Alignment In-the-Wild. Proceedings of the 2014 IEEE Conference on Computer Vision and Pattern Recognition (CVPR).

[15] Alabort-i-Medina J., Zafeiriou S. Bayesian Active Appearance Models. Proceedings of the 2014 IEEE Conference on Computer Vision and Pattern Recognition (CVPR).

[16] Hu Y., Zhang Y., Zhu Y., Cui R. (2010). AAM-based feature point extraction for pose-variant face. Comput. Eng. Appl..

[17] Fan X., Peng Q., Chen J.X., Xia X. (2009). An Improved AAM Fast Localization Method for Human Facial Features. J. Electron. Inf. Technol..

[18] Zhang J., Chen S. (2007). Delaunay Triangulation Algorithm in Planar Domain. Explos.-Proof Electr. Mach..

[19] Ke W., Chen Y., Pang Y., Li Q., Lu D. (2022). An activate appearance model-based algorithm for ear characteristic points positioning. Concurr. Comput. Pract. Exp..

[20] Chang M.L. (2017). Research on Facial Acupuncture Point Positioning Method Based on Feature Point Location Algorithm. Master’s Thesis.

[21] Wang Y., Jiang M., Huang N., Wen J. (2021). A location method of auricular acupoint divisions basedon active shape model algorithm. Beijing Biomed. Eng..

